# FFQ versus repeated 24-h recalls for estimating diet-related environmental impact

**DOI:** 10.1186/s12937-018-0425-z

**Published:** 2019-01-08

**Authors:** Elly Mertens, Anneleen Kuijsten, Johanna M. Geleijnse, Hendriek C. Boshuizen, Edith J. M. Feskens, Pieter van’t Veer

**Affiliations:** 1 Division of Human Nutrition and Health, Wageningen University, PO Box 8129, 6700 EV Wageningen, The Netherlands; 2 TiFN, Wageningen, P.O. Box 557, 6700 AN Wageningen, The Netherlands; 3 National Institute for Public Health and the Environment (RIVM), PO Box 1, 3720 BA Bilthoven, The Netherlands

## Abstract

**Background:**

There is an increasing interest in estimating environmental impact of individuals’ diets by using individual-level food consumption data. However, like assessment of nutrient intakes, these data are prone to substantial measurement errors dependent on the method of dietary assessment, and this often result in attenuation of associations.

**Purpose:**

To investigate the performance of a food frequency questionnaire (FFQ) for estimating the environmental impact of the diet as compared to independent 24-h recalls (24hR), and to study the association between environmental impact and dietary quality for the FFQ and 24hR.

**Methods:**

We analysed cross-sectional data from 1169 men and women, aged 20–76 years, who participated in the NQplus study, the Netherlands. They completed a 216-item FFQ and two replicates of web-based 24hR. Life cycle assessments of 207 food products were used to calculate greenhouse gas emissions, fossil energy and land use, summarised into an aggregated score, pReCiPe. Validity of the FFQ was evaluated against 24hRs using correlation coefficients and attenuation coefficients. Associations with dietary quality were based on Dutch Healthy Diet 15-index (DHD15-index) and Nutrient Rich Diet score (NRD9.3).

**Results:**

For pReCiPe, correlation coefficient between FFQ and 24hR was 0.33 when adjusted for covariates age, gender and BMI, and increased to 0.76 when de-attenuated for within-subject variation in the 24hR. Energy-adjustment slightly reduced these correlations (*r* = 0.71 for residuals of observed values and 0.59 for residuals of density values). Covariate-adjusted attenuation coefficient for the FFQ was 0.56 (ʎ_1_ = 0.56 and ʎ_1_ = 0.65 for observed and density residuals), slightly lower than without covariate adjustment. Diet-related environmental impact was inversely associated with the food-based DHD15-index for both FFQ and 24hR, while associations with the nutrient-based NRD9.3 were inconsistent.

**Conclusions:**

The FFQ slightly underestimated environmental impact when compared to 24hR. Associations with dietary quality are highly dependent on the diet score used, and less dependent on the method of dietary assessment.

**Electronic supplementary material:**

The online version of this article (10.1186/s12937-018-0425-z) contains supplementary material, which is available to authorized users.

## Background

Climate change has led to an increased interest in shifting towards environmentally-friendly food consumption patterns. Several studies have estimated the environmental impact related to dietary intake [1, 2]. This, however, is very challenging due to e.g.: high diversity in food products, their production practices, as well as inconsistencies in life cycle assessment (LCA) methods, including data availability and quality [3, 4]. On top of these, assessment of diet-related environmental impact depends on the method of dietary assessment, ranging from per capita food availability at the national level to food consumption at the individual level [5].

Assessment of the diet-related environmental impact was initially studied in the production domain dealing with a limited number of primary agricultural commodities of basic food items, using data on food availability, i.e. apparent food consumption data, defined as production – exports + imports, sourced from Eurostat and FAO databases. With the increasing availability of LCA data on single food products, it is now possible to study diet-related environmental impact in the consumer domain using food consumption data collected at the individual level. Moreover, individual-level dietary assessment allows combining environmental impact of the diet with other diet-related aspects, like dietary quality, acceptability of the diet, etc. [6]. So far, the few studies that have addressed this association with dietary quality used a multiple-day diet record [7–10] or a food frequency questionnaire (FFQ) [11, 12], but produced no clear results. Studies using diet records most often found that diet-related environmental impact was not associated with dietary quality [7–10], while an inverse association was reported in studies using FFQ [11, 12]. However, evaluation studies have shown that FFQs are subject to large between-person errors and introduce attenuation in associations with nutritional health outcomes [13, 14]. Moreover, as compared to 24hRs, FFQs are likely to perform less well for environmental impact as they purposively aggregate and incorporate food items that differentiate diets with respect to dietary quality rather than environmental impact. Until now, little is known about the potential influence of the method of dietary assessment on properly estimating diet-related environmental impact and its association with dietary quality.

Literature has acknowledged that all reported dietary intake values are prone to substantial measurement errors, both systematic, including intake-related and person-specific bias, and random errors, that often results in attenuation of the association [15]. In order to correct associations for dietary measurement error, a regression calibration approach, as introduced by Rosner et al. [16], is commonly used, which calculates attenuation coefficients in order to adjust for the bias caused by measurement error. Correct application of the regression calibration, however, is not guaranteed without a reference instrument that is unbiased and has errors independent of true exposure and independent of errors in dietary-reports [15, 17].

In the present study, we first evaluated the FFQ as a method to estimate environmental impact of individuals’ diets as compared to the 24hR as the individual-level and detailed reference method of dietary assessment. Second, we studied the association between food-based and nutrient-based diet scores based on 24hR and environmental impact based on either 24hR or FFQ with adjustment for random and systematic errors in assessment.

## Methods

### Study population

**Fig. 1:**
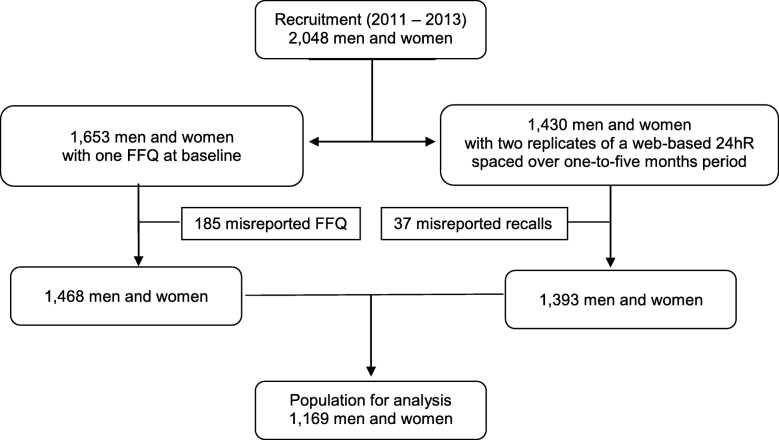
Flow diagram of subjects through the study

The present study was conducted with data obtained from the Nutrition Questionnaires plus (NQplus) study, conducted in Wageningen and its surroundings, the Netherlands [18, 19]. Initially, 2048 men and women, aged 20–70 years were recruited between 2011 and 2013. Subjects filled out an FFQ, general and health questionnaires, and underwent physical examinations at baseline, and multiple web-based recalls 24hRs were administered. Frequency of sampling 24hRs was not identical for each subject. Recall days were randomly selected and scheduled across the first year of the study with at least 40 days in between each other. Of the NQplus study population, a total of 1653 subjects completed one FFQ at a baseline and a total of 1430 subjects completed two replicates of a web-based 24hR spaced over one-to-five month period. We excluded 185 subjects with misreporting for the FFQ, and 37 subjects with misreporting for the 24hR. A total of 1169 subjects completed both an FFQ and two replicates of the 24hR, and remained for analysis (Fig. 1). The NQplus study was approved by the ethics committee of Wageningen University and conducted according to the Declaration of Helsinki, and all subjects provided their written informed consent.

### Methods of dietary assessment

The 24hR was a self-administered web-based highly-standardised version using the five-step multiple pass method, a validated technique to increase the accuracy of recalls [20]. Recall dates were randomly selected and scheduled evenly across the year and days of the week. For each subject, we included two recalls spaced over a one to five months period, resulting in 2338 recalls. Daily energy and nutrient intakes were calculated by multiplying the intake of food items with their nutrient content using the Dutch food composition table of 2011 [21].

The FFQ was developed to assess habitual intake, and consisted of 216 food items with questions on frequency and consumed amounts with a one-month reference period. This self-administered semi-quantitative FFQ was validated for energy intake [22], macronutrients, dietary fibre and selected micronutrients [23].

### Estimating diet-related environmental impact

Environmental impact was calculated based on LCA data from Blonk Consultants, available for 207 food products commonly consumed in the Dutch diet (Blonk Consultants data set version 2016) [24]. LCA were from cradle to grave, and included production, processing, packaging, transport, storage, preparation, cooking, avoidable waste and unavoidable food waste (inedible parts) at home, and waste incineration. Greenhouse gas emission (GHGE; in kilogram CO_2_-equivalents (kg CO_2_e)/day) covers carbon dioxide (CO_2_) emissions through the use of fossil fuels, methane (CH_4_) released during rearing of cattle and cultivation of certain crops, and nitrous oxide (N_2_O) released from fertilizers, manure and ploughing of grassland. Fossil energy use (FE, in Mega Joules (MJ)/day) covers the resources containing hydrocarbons needed for the production of food, and land use (LU, in m^2^*year/day) the surface needed for the production of food during a certain period of time. Environmental impact of the diet was reported for each impact category individually (i.e. GHGE, FE and LU), and aggregated - weighing their relative importance - into a single measure of environmental impacts, i.e. pReCiPe based on the principles of the ReCiPe method [25], calculated as

*pReCiPe* = 0.0459 ∗ *GHGE* + 0.0025 ∗ *FE* + 0.0439 ∗ *LU* where GHGE is greenhouse gas emissions in kilogram CO_2_ equivalents, FE fossil energy use in mega joules, LU land use in m^2^*year, and weighing values were obtained using a panel approach, then characterised and normalised using the year 2000 as reference year, and information was gathered for the European situation, as specified by the authors.

These LCA data were linked to food consumption data of the 24hRs and FFQ to calculate individual daily diet-related environmental impact using coding of the Dutch food composition table. For the 24hR, of the 1264 food products consumed in this cohort, 1198 (95%) food products were linked to LCA data either by direct matching or extrapolation. There was a direct match on food code for 203 (16%) food products consumed in this cohort, which covered 50% of total food weight intake, excluding beverages, and 53% of total energy intake. Extrapolations were made to other food products consumed according to the 24hR based on similarities in type of food product (11%) or production method (56%), and based on ingredient composition by using standard recipes for composite foods (12%). For the FFQ, the 216 FFQ-items were disaggregated into 1159 food products with different contribution percentages based on Dutch dietary survey data, coded by the Dutch food composition table, and subsequently matched with LCA data on their food code. When LCA data were not available for all food products within an FFQ-item (*n* = 135), we scaled the food products with LCA data in such a way that the FFQ-item was 100% represented by those food products, while accounting for their contribution percentage. LCA data were available for 167 FFQ-items covering 89% of the total food weight and 86% of the total energy intake. Remaining FFQ-items (*n* = 49) received an extrapolated value based on similarities in type of food product, production method and ingredient composition.

### Estimating dietary quality

Dietary estimates of the 24hR were analysed for their dietary quality using a diet score based on food groups, i.e. the Dutch Healthy Diet Index 2015 (DHD15-index) [26], and one based on nutrients, i.e. the Nutrient Rich Diet score (NRD9.3) [10, 27]. DHD15-index consists of fifteen food groups included the Dutch food-based dietary guidelines of 2015: vegetables, fruit, wholegrain products, legumes, nuts, dairy, fish, tea, fats and oils, filtered coffee, red meat, processed meat, sweetened beverages and fruit juices, alcohol, and salt. A proportional score between 0 and 10 was assigned to all other food groups, and the final score was the mean of all food groups and ranged from 0 (minimal adherence) to 10 (maximal adherence). NRD9.3 was based on the principles of the Nutrient Rich Food Index, NRF9.3 [28, 29]. This NRF9.3 algorithm is the unweighted sum of percentage daily values (DVs) for nine nutrients to encourage (protein, dietary fibre, calcium, iron, potassium, magnesium, and vitamin A, C and E) minus the sum of percentage maximum recommended values for three nutrients to limit (saturated fat, added sugar, and sodium), calculated per 100 kcal and capped at 100%DV. We expressed nutrient intakes relative to a daily energy intake of 2500 kcal for men and of 2000 kcal for women to obtain a daily nutrient density score.

### Covariates

Data were collected on age (years), sex, educational level (low: no, lower or lower vocational education; intermediate: intermediate vocational; and high: higher vocational or university), smoking status (never/former/current) by means of questionnaires. Physical activity was assessed using the Short QUestionnaire to Asses Health enhancing physical activity (SQUASH) [30], and was categorised according to the average time spent per week doing commuting, leisure-time and household activities, and activities at work (Metabolic Equivalent of Task (MET) in minutes per week); low: < 500; moderate: 500 ≤ MET< 1000; high: MET ≥1000). Body weight was measured by a trained research assistant without shoes and heavy clothing and with empty pockets on a digital scale (SECA 877; SECA Corp.), and height was measured without shoes using a stadiometer (SECA 213; SECA Corp.). Body Mass Index (BMI) was calculated as body weight (kg) divided by height squared (m^2^).

### Measurement error model

It was assumed that estimates obtained from 24hRs were the best available standards to approximate true diet-related environmental impact, as no independent reference methods are available [31, 32]. In contrast, in the FFQ, constant bias at the group level, intake-related bias and person-specific bias were assumed to be present. The measurement error model was specified as:

1 $$ {\displaystyle \begin{array}{cc}24\hbox{-} \mathrm{hour}\ \mathrm{recall}\ (R):& R=T+{e}_R\\ {}\mathrm{FFQ}\ (Q):& Q={A}_Q+{B}_QT+q+{e}_Q\end{array}} $$

where T is the true (unknown) intake, e the within-person random error, and A the overall constant bias at group level, B the intake-related bias and q the person-specific bias for the FFQ. By this model, it was assumed that estimates from two replicates of the 24hRs are statistically independent and contains no intake-related bias and no person-specific bias [33].

### Statistical methods

To evaluate the performance of the FFQ versus the 24hR, linear mixed models with a random intercept for subjects were applied to account for the two replicates of the 24hRs per subject. Attenuation coefficient was estimated as the slope in the linear regression of the reference method (i.e. 24hR) on the FFQ through the following linear mixed model:

2 $$ {R}_{ij}={\lambda}_0+\lambda {{}_1Q}_i+{\mu}_i+{e}_{ij} $$

where R_ij_ is the j^th^ observation of the recall for the i^th^ individual, Q_i_ the FFQ-report of that individual, u_j_ the random intercept for that individual and e_ij_ the random within-person variation, λ_0_ is the method-specific intercept and λ_1_ the attenuation coefficient. The random terms were assumed to be independent, normally distributed with mean zero and variances σ^2^(u) and σ^2^(e). Correlation coefficients between FFQ and the average of two 24hRs were estimated as Pearson correlations, without and with adjustment for covariates age, gender and BMI. To account for within-subject variation in the 24hR, correlation coefficients were de-attenuated by dividing by the square root of the intra-class correlation coefficient (ICC) of the replicates of the 24hR; ICC was calculated as the variance in random intercept divided by the total variance obtained from a mixed model without Q as covariate under the assumption of no person-specific bias [34].

In the analysis of diet associations, e.g.: impact vs quality, covariate adjustment is essential for the internal study validity, hence the usual covariates age (continuous), gender (men/women), and BMI (continuous) were included in the calibration equation [31]. In addition, stratified analyses were performed for men and women separately (results in Additional files 1 and 2). Specific attention was paid to energy intake as a key covariate in diet analyses, using linear regression of diet-related environmental impact on energy-intake [35]; the latter was done for both observed values and densities, i.e. observed values divided by total energy intake, and standardised to 2000 kcal. Densities and residuals were calculated for each method of dietary assessment using estimates as measured by that method of dietary assessment.

To illustrate the possible influence of the method of dietary assessment, we analysed the association between dietary quality and diet-related environmental impact by linear regression analyses with adjustments for age, gender, BMI, and energy intake. Dietary quality was assessed by the food-based DHD15-index and the nutrient-based NRD9.3 both based on the 24hR as the alleged gold standard reference. When the (explanatory) diet-related environmental impact variables were derived from the 24hR the associations with diet scores were corrected for within-subject variation using Best Linear Unbiased Predictions (BLUPs) from a mixed model without Q as covariate [36]. When the diet-related environmental impact variables were based on the FFQ, the association with dietary quality was calibrated using a mixed model accounting for random effects (i.e. the predicted values from eq. 2 with covariates added). All statistical analyses were performed using SAS version 9.3 (SAS Institute, Inc.).

## Results

Mean age of the population was 53 (SD 12) years and mean BMI was 25.6 (SD 3.7) kg/m^2^ (Table 1). More than 60% of the population completed a level of higher education, less than 35% had a high level of physical activity and less than 10% was current smoker. Approximately half of the population (48%) were women, who were on average younger, had a lower BMI, a lower level of physical activity, and a lower energy intake than men. Mean diet scores, measured by 24hR, were 4.9 (SD 1.0) for DHD15-index and 500 (SD72) for NRD9.3; with the diets of women having a higher dietary quality (respectively 5.3 vs 4.6, and 507 vs 493). Measured by two replicates of the 24hR, mean (SD) estimated crude environmental impact of the diet was 3.6 (SD 1.5) kg CO2e/d for GHGE, 31.1 (SD 9.2) MJ/d for FE, and 4.2 (SD 1.8) m^2^*year/d for LU; summarised in a pReCiPe of 0.43 (SD 0.16), with the diets of women having a lower environmental impact (pReCiPe of 0.39 versus 0.46).

**Table 1:** General characteristics of the NQplus study (*n* = 1169)^a^

	Total (*n* = 1169)		Men (*n* = 606)		Women (*n* = 563)	
Age, years	53.2	(11.5)	55.6	(10.7)	50.6	(11.7)
BMI, kg/m^2 b^	25.6	(3.7)	26.2	(3.3)	24.9	(3.9)
Education level ^c^						
Low	67	(6%)	46	(7%)	21	(4%)
Intermediate	343	(29%)	162	(27%)	181	(32%)
High	757	(65%)	397	(66%)	360	(64%)
Physical activity						
Low	539	(46%)	249	(41%)	290	(51%)
Moderate	224	(19%)	114	(19%)	110	(20%)
High	406	(35%)	243	(40%)	163	(29%)
Smoking status ^d^						
Never	587	(53%)	263	(45%)	324	(61%)
Former	435	(39%)	259	(45%)	176	(33%)
Current	90	(8%)	56	(10%)	34	(6%)
Energy intake, kcal/d ^e^	2012	(583)	2200	(617)	1808	(466)
DHD15-index ^e^	4.92	(1.00)	4.61	(0.94)	5.29	(0.96)
NRD9.3 ^e^	500	(72)	493	(71)	507	(73)
GHGE, kgCO_2_e/d ^e^	3.64	(1.46)	3.94	(1.60)	3.32	(1.20)
FE, MJ/d ^e^	31.10	(9.20)	33.36	(9.83)	28.66	(7.77)
LU, m^2^*year/d ^e^	4.15	(1.82)	4.57	(1.99)	3.71	(1.51)
pReCiPe ^e^	0.43	(0.16)	0.46	(0.18)	0.39	(0.14)

Abbreviations: DHD15-index, Dutch Healthy Diet Index 15; NRD9.3, Nutrient Rich Diet score 9.3;GHGE, greenhouse gas emissions; FE, fossil energy use; LU, land use; pReCiPe, a weighted summary score for GHGE, FE, and LU

^a^Values are expressed as mean (standard deviations), numbers and percentages. Comparisons between men and women were tested by independent samples t-test for continuous variables and chi-square test for categorical variables. All characteristics above were statistically significant using *P*-value below 0.05. ^b^Data were available for 1168 subjects, i.e. 605 men and 563 women. ^c^Data were available for 1167 subjects, i.e. 605 men and 562 women. ^d^Data were available for 1112 subjects, i.e. 578 men and 534 women. ^e^ Dietary estimates were crude values based on two 24-h recalls

Meat, dairy, and beverage consumption contributed the most to the environmental impact, irrespective of the method of dietary assessment (meat 29% of total daily dietary pReCiPe, dairy 16% and beverages 15% according to 24hR, and with similar values for the FFQ) (Table 2). Impacts of type of meat, however, differed by method of dietary assessment with for the FFQ a higher contribution to pReCiPe and its components from non-processed meat and a lower contribution from processed meat (18% vs 9%) as compared to the 24hR (15% vs 14%); consistent with reported intake differences. In addition, reported intakes of dairy and plant-based foods, like potatoes, bread, vegetables, legumes and fruit, were in general higher for the FFQ than for the 24hR. Contribution of the different food groups to daily diet-related environmental impact was dependent on the environmental impact measures for some food groups; meat had a higher share in total daily dietary GHGE and LU than in FE, while the opposite was seen for plant-based foods, fish and beverages.

**Table 2:** Contribution of the different food groups to daily intake and environmental impact in the NQplus Study, using FFQ and 24-h recall

Food groups	24-h Recall						FFQ					
	g/d (%)	E%d (%)	GHGE (%)	FE (%)	LU (%)	pReCiPe (%)	g/d (%)	E%d (%)	GHGE (%)	FE (%)	LU (%)	pReCiPe (%)
Potatoes	2.5	4.1	1.7	3.0	2.1	2.1	3.3	3.9	2.0	3.5	2.5	2.5
Cereals, cereal products												
Bread products	5.2	18.1	3.7	5.5	4.4	4.3	5.5	15.2	3.4	5.1	4.3	4.1
Cake, Biscuits	1.5	7.4	2.6	2.8	3.1	2.9	1.5	6.2	2.1	2.5	2.9	2.5
Pasta,rice,couscous	2.0	5.4	1.8	1.8	2.9	2.3	3.0	5.5	2.7	2.2	3.7	3.1
Vegetables	5.1	1.9	5.1	8.0	1.9	4.3	6.9	2.3	5.4	9.1	2.0	4.6
Legumes	0.2	0.4	0.2	0.4	0.2	0.3	0.6	0.7	0.6	0.9	0.4	0.6
Fruit	5.7	5.1	3.8	5.1	1.9	3.3	8.0	5.4	4.9	6.7	2.4	4.2
Nuts and seeds	0.4	3.3	0.6	0.6	2.3	1.4	0.6	4.4	0.9	0.9	3.3	1.9
Dairy												
Cheese	1.2	5.8	8.7	4.7	5.6	6.6	1.3	4.8	8.4	4.5	5.3	6.4
Milk^a^	6.1	4.2	5.9	4.9	4.0	4.9	7.8	4.1	6.8	5.5	4.5	5.6
Milk-based desserts^b^	4.4	4.7	5.4	4.3	3.3	4.3	5.4	4.8	5.8	4.7	3.5	4.6
Meat												
Non-processed^c^	1.4	3.1	15.7	9.4	16.9	15.0	1.6	2.8	18.6	9.8	20.5	17.8
Processed^d^	1.7	5.5	14.9	8.8	14.3	13.6	1.4	3.7	9.5	6.2	8.8	8.6
Fish	0.7	1.7	2.9	4.9	1.1	2.5	0.7	1.2	3.0	5.1	0.6	2.3
Eggs	0.5	1.0	1.3	1.5	1.6	1.5	0.6	0.9	1.4	1.6	1.7	1.6
Vegetarian products												
Soy drink, desserts^e^	0.1	0.1	0.0	0.1	0.1	0.0	0.3	0.2	0.1	0.1	0.1	0.1
Meat replacers	0.2	0.3	0.2	0.3	0.2	0.2	0.2	0.3	0.3	0.4	0.3	0.3
Fats, Oils, Sauces	1.1	6.4	2.5	2.2	4.3	3.2	1.8	10.1	3.2	3.3	7.4	5.0
Sugar, Sweets^f^	0.9	5.6	1.1	1.7	1.3	1.3	1.8	7.5	3.4	2.6	3.5	3.3
Snacks	0.5	2.7	1.5	1.7	1.8	1.7	1.3	5.7	2.9	3.4	4.4	3.6
Soup, Composite dishes	4.0	4.4	7.8	7.3	12.3	9.6	2.7	3.3	3.1	1.8	3.7	3.3
Beverages												
Non-alcoholic	48.0	2.8	7.9	13.6	8.3	9.1	40.0	4.7	9.9	16.4	13.3	12.5
Alcoholic	6.2	5.6	4.5	7.3	6.0	5.6	3.7	2.2	1.6	2.6	1.0	1.5

DHD15, Dutch Healthy Diet Index 15; NRD7.3, Nutrient Rich Diet score 7.3; GHGE, greenhouse gas emissions; FE, fossil energy use; LU, land use; pReCiPe, a weighted summary score for GHGE, FE, and LU

^a^ milk: milk, milk beverages (chocolate milk) and coffee milk ^b^ milk-based desserts: all kind of yoghurts, creams, and milk-based puddings and dessert ^c^ non-processed meat: beef, pork, and chicken ^d^ processed meat: meat products as sandwich filling, ham, ready-to-eat minced meat, sausages, organ meat and miscellaneous types ^e^ soy drinks, desserts: soy-based drinks, yoghurts, puddings and creams ^f^ Sugar, sweets: sugar, candy, sweet and savoury sandwich filling like jams, honey, chocolate spread, peanut butter

Table 3 shows the diet-related environmental impact according to the FFQ and the 24hR as well as the ICC for the latter. Observed values for FFQ and 24hR were similar for protein, and environmental indicators (< 5% difference), but energy intake was overestimated by the FFQ (6%). After energy-adjustment, we observed underestimated values for protein intake (6%) and for diet-related environmental impact measures (7–10%) by the FFQ. ICC for replicates of the 24hR were low (≤ 0.30) for all variables under study; they were slightly lower for observed residuals (0.12–0.22) than for observed values (0.17–0.28) and density residuals (0.19–0.30). Thus, most of the observed variation was due to within-person variation, such as day-to-day variability, rather than between-person variation.

**Table 3:** Diet-related environmental impact according to the food frequency questionnaire (FFQ) and two replicates of the 24-h recall (24hR, with intra-class correlation coefficient) and group level bias, with correlation between the methods (crude, adjusted, de-attenuated) and attenuation coefficient (crude, adjusted)^a^ for observed and energy-adjusted values standardised to a 2000 kcal diet

	FFQ		2 replicates of 24hR				Correlation coefficient (24hR with FFQ)						Attenuation coefficient λ_1_			
Dietary variables	Mean	(SD)	Mean	(SD)	ICC	%bias	Crude (95%CI)		Adjusted^b^(95%CI)		De-attenuated (95%CI)		Crude (SE)		Adjusted^b^ (SE)	
*Observed values*																
Energy, kcal/d	2139	(532)	2012	(583)	0.31	6.3	0.47	(0.43; 0.52)	0.38	(0.33; 0.43)	0.68	(0.59; 0.77)	0.52	(0.03)	0.42	(0.03)
Protein, g/d	77.6	(18.5)	78.2	(23.5)	0.27	−0.8	0.46	(0.41; 0.50)	0.39	(0.34; 0.44)	0.75	(0.65; 0.84)	0.58	(0.03)	0.51	(0.04)
GHGE, kgCO_2_e/d	3.50	(0.87)	3.64	(1.46)	0.21	−3.8	0.35	(0.30; 0.40)	0.30	(0.25; 0.35)	0.66	(0.54; 0.77)	0.59	(0.05)	0.53	(0.05)
FE, MJ/d	30.19	(6.71)	31.10	(9.20)	0.28	−2.9	0.45	(0.40; 0.49)	0.40	(0.35;0.44)	0.75	(0.66; 0.84)	0.62	(0.04)	0.57	(0.04)
LU, m^2^*year/d	4.01	(1.08)	4.15	(1.82)	0.17	−3.4	0.37	(0.32; 0.42)	0.31	(0.26; 0.36)	0.75	(0.62; 0.87)	0.63	(0.05)	0.56	(0.05)
pReCiPe	0.41	(0.10)	0.43	(0.16)	0.19	−4.7	0.39	(0.34; 0.43)	0.33	(0.28; 0.38)	0.76	(0.64; 0.87)	0.62	(0.04)	0.56	(0.05)
*Energy-adjusted values by regression residuals of observed values on energy (observed residuals)*																
Protein, g/d	73.5	(10.3)	77.9	(14.2)	0.20	−5.6	0.35	(0.30; 0.40)	0.33	(0.28; 0.38)	0.75	(0.63; 0.86)	0.49	(0.04)	0.46	(0.04)
GHGE, kgCO_2_e/d	3.33	(0.61)	3.63	(1.24)	0.12	−8.3	0.26	(0.20; 0.31)	0.23	(0.17; 0.28)	0.66	(0.50; 0.81)	0.52	(0.06)	0.48	(0.06)
FE, MJ/d	28.82	(4.21)	30.98	(6.77)	0.22	−7.0	0.39	(0.34; 0.43)	0.35	(0.30; 0.40)	0.76	(0.65; 0.86)	0.62	(0.04)	0.59	(0.05)
LU, m^2^*year/d	3.80	(0.71)	4.14	(1.56)	0.13	−8.2	0.30	(0.24; 0.35)	0.27	(0.21; 0.32)	0.73	(0.58; 0.88)	0.65	(0.06)	0.60	(0.06)
pReCiPe,	0.39	(0.07)	0.43	(0.14)	0.14	−9.3	0.30	(0.24; 0.35)	0.26	(0.21; 0.32)	0.71	(0.57; 0.85)	0.61	(0.06)	0.56	(0.06)
*Energy-adjusted values by regression residuals of densities on energy (density residuals)*																
Protein density, %	14.9	(2.0)	15.9	(3.1)	0.20	−6.3	0.37	(0.31; 0.41)	0.35	(0.29; 0.40)	0.78	(0.66; 0.89)	0.57	(0.04)	0.54	(0.04)
GHGEdensity,kgCO_2_e/d	3.38	(0.60)	3.73	(1.30)	0.19	−9.4	0.28	(0.23; 0.33)	0.25	(0.20; 0.30)	0.59	(0.46; 0.71)	0.61	(0.06)	0.57	(0.06)
FE density, MJ/d	29.31	(4.09)	32.14	(7.62)	0.30	−8.8	0.39	(0.34; 0.44)	0.36	(0.31; 0.41)	0.66	(0.56; 0.74)	0.73	(0.05)	0.69	(0.05)
LU density, m^2^*year/d	3.83	(0.70)	4.24	(1.72)	0.21	−9.7	0.30	(0.24; 0.35)	0.27	(0.21; 0.32)	0.59	(0.47; 0.71)	0.73	(0.07)	0.67	(0.07)
pReCiPe density	0.40	(0.06)	0.44	(0.15)	0.22	−9.1	0.31	(0.26; 0.36)	0.28	(0.22; 0.33)	0.59	(0.48; 0.71)	0.70	(0.06)	0.65	(0.07)

ICC, intra class correlation coefficient; GHGE, greenhouse gas emissions; FE, fossil energy use; LU, land use; pReCiPe, a weighted summary score for GHGE, FE, and LU; % bias, group-level bias calculated as (mean intake FFQ /mean intake 24hR)×100; 100; correlation coefficient (95%CI) estimated as the Pearson correlation coefficient; de-attenuated correlation coefficient (95%CI) estimated as the Pearson correlation coefficient/√ICC_24hR_; Attenuation coefficient λ_1_ (SE) estimated as the slope in the linear regression of the 24hR on the FFQ using linear mixed models to account for within-person day-to-day variability

^a^Mean values with their standard deviations, correlation coefficient with its 95% confidence intervals, attenuation coefficient with its standard error

^b^Adjusted for age, gender and BMI

The crude correlation coefficient between FFQ and 24hR was 0.46 for protein, and ranged from 0.35 for GHGE to 0.45 for FE, but weakened after covariate adjustment. When accounting for random error in the 24hR, the correlation coefficient was 0.75 for protein, and ranged from 0.66 for GHGE to 0.76 for pReCiPe, as shown by the de-attenuated correlation coefficient. After adjustment for energy, de-attenuated correlation coefficients were similar when using observed residuals, but they were lower when using density residuals, except for protein.

Estimated attenuation coefficients, as displayed by the regression slopes λ_1_, were all below one, pointing to a flattened slope phenomenon in associations when using the FFQ. This attenuation appeared to be more severe with the inclusion of the covariates age, gender and BMI in the measurement error model for all variables under study (attenuation coefficients were lower). Covariate-adjusted attenuation coefficient for observed values was 0.51 for protein, and ranged from 0.53 for GHGE to 0.57 for FE. Energy-adjustment by the residual method of observed values showed similar attenuation coefficients as with non-energy-adjusted values; and for density residuals, the fully-adjusted attenuation coefficients tended to be higher, i.e. attenuation was lower than for the non-energy adjusted values, but less marked for protein and GHGE (with attenuation coefficient of 0.54 for protein and from 0.57 for GHGE to 0.69 for FE).

In stratified analysis, patterns of results for group-mean bias, correlation coefficients, and attenuation coefficients were generally similar for men and women (Additional files 1 and 2). Estimated correlation coefficients and attenuation coefficients for observed values did not change with covariate adjustment; indicating that gender explained most of the variation in this population. However, when using energy-adjusted values, as compared to non-energy-adjusted values, attenuation coefficients appeared to be higher for density residuals, and this was more marked in women than in men.

Table 4 shows the association between dietary quality (DHD15-index and NRD9.3) and diet-related environmental impact using observed and de-attenuated 24hR-values, and observed and calibrated FFQ-values, for different methods of energy adjustment. Regression coefficients represent the percentage change in diet score per unit increase in diet-related environmental impact. Diet-related environmental impact was significantly inversely associated with the food-based DHD15-index, for all environmental impact measures, and for all methods of dietary assessment. Compared to de-attenuated 24hR-values, regression coefficients using FFQ-values as observed were weakened, and became closer when calibrated FFQ-values were used. For the nutrient-based NRD9.3, no statistically significant associations were observed for summary score pReCiPe and its component GHGE, but a positive significant association was observed for FE and a significant negative association for LU. Using de-attenuated 24hR-values showed a negative association for LU, while using FFQ-values as observed showed a positive association and calibration could repair this. Considering the method of energy adjustment, for both DHD15-index and NRD9.3, associations based on de-attenuated 24hR-values were stronger for observed residuals than for observed values with inclusion of energy in the multivariate model, but were weaker for density residuals.

**Table 4:** Regression coefficients (with 95% confidence intervals)^a^ for dietary quality, measured by food-based DHD15 -index and nutrient-based NRD9.3, and diet-related environmental impact, measured by GHGE, FE, LU, and pReCiPe, using observed and de-attenuated 24-h recall (24hR)-values, and observed and calibrated FFQ-values, for different methods of energy adjustment in the total population

Response variables	DHD15-index (based on 2 replicates of 24hR)						NRD9.3 (based on 2 replicates of 24hR)					
Explanatory variables	Observed values		Observed residuals		Density residuals		Observed values		Observed residuals		Density residuals	
	β	(95%CI)	β	(95%CI)	β	(95%CI)	β	(95%CI)	β	(95%CI)	β	(95%CI)
GHGE, per 1 kgCO2e/d												
24hR as observed	-3.3	(-4.1; -2.4)	-3.3	(-4.1; -2.4)	-2.7	(-3.5; -1.8)	-0.2	(-0.9; 0.6)	-0.2	(-0.9; 0.6)	-0.4	(-1.1; 0.3)
De-attenuated 24hR	-11.3	(-14.1;-8.4)	-14.5	(-18.0;-10.9)	-8.3	(-10.8; -5.8)	-0.5	(-3.1; 2.0)	-0.7	(-4.0; 2.7)	-1.2	(-3.3; 1.0)
FFQ as observed	-3.9	(-5.7; -2.0)	-3.9	(-5.7; -2.0)	-3.9	(-5.8; -2.0)	1.0	(-0.5; 2.5)	1.0	(-0.5; 2.5)	1.1	(-0.5; 2.6)
Calibrated FFQ	-8.3	(-10.8;-5.8)	-10.8	(-13.6; -8.0)	-7.7	(-9.8; -5.5)	0.9	(-1.2; 3.2)	0.8	(-1.7; 3.4)	-0.1	(-2.0; 1.8)
FE, per 5 MJ/d												
24hR as observed	-1.3	(-2.1; -0.5)	-1.3	(-2.1; -0.5)	-0.8	(-1.6; -0.1)	1.3	(0.7; 2.0)	1.3	(0.7; 2.0)	0.7	(0.1; 1.3)
De-attenuated 24hR	-2.9	(-4.8; -1.1)	-3.6	(-5.8; -1.3)	-1.8	(-3.4; -0.2)	3.1	(1.5; 4.6)	2.3	(1.9; 5.7)	1.5	(0.2; 2.7)
FFQ as observed	-1.7	(-3.1; -0.3)	-1.7	(-3.1; -0.3)	-1.6	(-3.0; -0.2)	2.3	(1.2; 3.4)	2.3	(1.2; 3.4)	2.4	(1.2; 3.5)
Calibrated FFQ	-2.5	(-4.3; -0.8)	-3.1	(-5.0; -1.3)	-1.9	(-3.3; -0.5)	3.6	(2.1; 5.0)	3.8	(2.3; 5.4)	2.1	(0.9; 3.3)
LU, per 1 m^2^*year/d												
24hR as observed	-3.2	(-3.9; -2.5)	-3.2	(-3.9; -2.5)	-2.6	(-3.2; -2.0)	-0.6	(-1.2; -0.1)	-0.6	(-1.2; -0.1)	-0.9	(-1.4; -0.4)
De-attenuated 24hR	-10.4	(-12.5; -8.3)	-13.2	(-15.8;-10.6)	-7.4	(-9.1; -5.6)	-2.1	(-4.0; -0.2)	-2.7	(-5.1; -0.3)	-2.6	(-4.1; -1.1)
FFQ as observed	-6.3	(-7.8; -4.7)	-6.3	(-7.8; -4.7)	-6.2	(-7.7; -4.7)	0.2	(-1.1; 1.5)	0.2	(-1.1; 1.5)	0.1	(-1.2; 1.4)
Calibrated FFQ	-9.6	(-11.4; -7.7)	-11.4	(-13.3; -9.4)	-8.0	(-9.4; -6.4)	-0.8	(-2.4; 0.9)	-0.9	(-2.7; 0.9)	-1.7	(-3.0; -0.4)
pReCiPe, per 0.1												
24hR as observed	-3.3	(-4.1; -2.5)	-3.3	(-4.1; -2.5)	-2.6	(-3.3; -1.9)	-0.3	(-0.9; 0.4)	-0.3	(-0.9; 0.4)	-0.6	(-1.2;0.0)
De-attenuated 24hR	-10.0	(-12.3; -7.7)	-12.9	(-15.7; -9.9)	-7.1	(-9.1; -5.2)	-0.8	(-2.9; 1.2)	-1.1	(-3.7; 1.6)	-1.6	(-3.3; 0.0)
FFQ as observed	-5.1	(-6.8; -3.4)	-5.1	(-6.8; -3.4)	-5.1	(-10.3; -5.1)	0.9	(-0.5; 2.3)	0.9	(-0.5; 2.3)	0.9	(-0.5; 2.4)
Calibrated FFQ	-8.6	(-10.7; -6.5)	-10.6	(-12.8; -8.3)	-7.3	(-9.0; -5.5)	0.5	(-1.3; 2.4)	0.4	(-1.6; 2.5)	-0.6	(-2.1; 0.9)

DHD15-index, Dutch Healthy Diet Index 15; NRD9.3, Nutrient Rich Diet score 9.3; GHGE, greenhouse gas emissions; FE, fossil energy use; LU, land use; pReCiPe, a weighted summary score for GHGE, FE, and LU. De-attenuated 24hR-values estimated using the method of Best Linear Unbiased Prediction (BLUP) to correct for random error. Calibrated FFQ values calculated as the predicted values from a mixed model with FFQ-values, age, gender and BMI as covariates, accounting for random effects

^a^ Regression coefficients represent the percentage change in diet score per unit increase in diet-related environmental impact, and are adjusted for energy intake (continuous and using estimates as measured by that method of dietary assessment), age (continuous), gender (men/women), and BMI (continuous)

## Discussion

Group-mean differences between FFQ and the reference 24hR were small (< 5%) for absolute values of GHGE, FE, LU and pReCiPe. Covariate-adjusted de-attenuated correlation coefficients between FFQ and 24hR were around 0.70, and attenuation coefficients were around 0.55 for observed values on diet-related environmental impact measures. When we studied the association between environmental impact and dietary quality, an inverse association was observed when dietary quality was assessed using a food-based score (DHD15-index), but inconsistent and weak associations were seen when using a nutrient-based score (NRD9.3).

To the best of our knowledge, this is the first calibration study on diet-related environmental impact measures comparing the environmental impact obtained from FFQ with that of the 24hR. The latter was used as reference instrument since no truly gold standard exist. As a means for comparison, we calculated correlation coefficients and attenuation factors for protein intake as this is a widely studied nutrient in dietary validation studies. Correlation coefficients and attenuation coefficients for intake of energy and protein are in line with earlier calibration studies [33, 37]. In the present study, the unadjusted correlation coefficient for protein was 0.46 (men: 0.41; women: 0.38), and the unadjusted attenuation coefficient for protein intake was 0.58 (men; 0.54; women. 0.48). Pooled analysis of protein intake in eight European validation studies within the European Prospective Investigation into Cancer [37] reported correlation coefficients between FFQ and 24hR varying between 0.35 and 0.67, and attenuation coefficients for the FFQ on 24hR between 0.26 and 0.63. In the US, the Observing Protein and Energy Nutrition (OPEN) study [33] reported correlation coefficients of 0.31 for men and 0.33 for women and attenuation coefficients of 0.53 for men and 0.70 for women. Thus, as compared to protein, correlation coefficients between these two methods tended to be slightly lower for all diet-derived measures of environmental impact, whereas attenuation coefficients were slightly higher, especially for FE and LU. As there was a strong correlation between measure of environmental impact and protein intake (correlation coefficients between 0.6–0.9), results might to some extent be affected by protein-poor food sources that contributed to diet-related environmental impact with their intake and contribution highly varying by the method of dietary assessment.

Changes in dietary intake are generally based on iso-caloric exchanges of foods, hence the need to keep energy intake constant when comparing diets between groups. Previous studies on the measurement error structure of self-reported protein intake have noted that the attenuation is less severe when energy intake is taken into account by either regression of protein intake on energy intake (protein residuals) or by the density method (dividing energy from protein intake by energy) [13, 33]. Our analysis shows that the same holds for diet-derived measures of environmental impact, with less attenuation for density residuals than for observed residuals. This is in line with the results of Table 4: regression coefficients using observed FFQ-values were closer to those using de-attenuated 24hR-vales for densities residuals than for observed residuals. Measurement errors in the assessment of environmental impact are strongly correlated with errors in the measurement of total energy intake, and this appeared to be more marked for observed residuals, as shown by the lower ICC. This finding further supports the importance for using energy-adjusted intakes in nutritional epidemiology, however caution must be applied for their interpretation, as has been discussed previously [35]. Diet-related environmental impact is preferably expressed in relative values (i.e.: impact per 2000 kcal) rather than absolute values, because of the application of densities in public health recommendations. Individuals and populations can reduce their diet-related environmental impact per kcal consumed by replacing the intake of specific foods by environmental-friendly alternatives, thus by changing diet composition rather than total energy intake, unless physical activity and body weight have been changed substantially. Total energy intake is however strongly positively related to diet-related environmental impact as observed, which are absolute impact levels important in environmental sciences, hence the need for using density residuals.

In our study, the assessment of environmental sustainability of the diet was restricted by the availability of LCA data from 207 food products, resulting in an imprecise estimation of the environmental impact of the diet for both FFQ and 24hR. In addition, methods of dietary assessment to date have been developed to monitor food and nutrient intakes, without considering sustainable dietary practices, such as food origin, packaging and preparation methods, transport, storage, food waste, etc. Our results, however, show that the measurement errors for LCA-based environmental impact measures are of similar size as protein intake, which is at the better end of the range of errors in assessment of food and nutrient intake [33, 37, 38]. This was not hypothesized a priori. Nutrient-based selection of food items does not necessarily capture the variation for diet-related environmental impact measures, but apparently it does for the 24hR and FFQ in this study. This suggests that errors in classification (foods vs grouped items), portions size (specific vs standard) and frequency (FFQ only) largely explain the differences between the 24hR and FFQ, and eventually result in similar errors for estimated daily nutrient intake and environmental impact. Still, 24hRs (and diet records) provide more objective data on dietary practices, and for some food products packaging and preparation methods might by this time be recorded dependent on the dietary knowledge level and cooking skills of the subject. Provided that LCA data are more widely available for all kind of food products, these open-ended methods of dietary assessment that consider both healthy and environmental dietary practices would perform much better as compared to the FFQ, unless specifically designed for assessing environmental impact.

The secondary aim of this paper was to investigate the association between dietary quality (DHD15-index and NRD9.3) and environmental impact of the diet (24hR-based or FFQ-based). Dietary quality was used as independent variable using the 24hR, and measures of environmental impact as dependent variable using both methods of dietary assessment (24hR and FFQ) without and with accounting for measurement error. Differences in regression coefficients can therefore be attributed to the ability of the 24hR versus FFQ to assess associations with environmental impact. Our results show that quality of the food pattern (DHD15-index in our case) is similarly related to all environmental impact measures under study, and more environmentally-friendly diets (lower value) tend to score better on food-based dietary quality (hence a negative regression coefficient); this is irrespective of the environmental impact measures. However, when nutrient quality of the diet (NRD9.3 in our case) is considered, the results differ by environmental impact measure and whether 24hR or FFQ was used as the method of dietary assessment.

In the detail for NRD9.3, we showed that nutrient quality tended to be positively associated with diet-related FE; but inversely with diet-related GHGE and LU. The reason for these apparently conflicting findings is likely attributable to the contribution of different food groups to daily diet-related environmental impact and nutrient intake. The positive association for diet-related FE with NRD9.3 is likely to be driven by food sources such as fish, bread, fruit and vegetables that have a higher contribution to total-diet related FE as compared to GHGE and LU (Table 2). Moreover, these foods have a high nutrient density contributing to high intakes of dietary fibre, potassium, magnesium, iron, vitamin C, E, and low intakes of sodium, added sugar and saturated fat. In contrast, the inverse association for LU is likely to be driven by the low contribution of fruit and vegetables to diet-related LU as compared to GHGE and FE (Table 2). This inverse association between LU and NRD9.3 was seen when using a 24hR, but not when using an FFQ; which might be explained by the higher intakes of fruit and vegetables observed in the FFQ. As the abovementioned foods played a less important role in the DHD15-index (only four out of fifteen components), an inverse association with diet-related environmental impact was found for this food-based diet score.

Our results are supported by previous studies that also showed inverse associations between diet-related GHGE and the food-based scores [11, 12, 39], whereas studies using nutrient-based scores showed no clear associations [7, 8, 40]. This discrepancy between results for food-based scores and nutrient-based scores may be explained by the different components included in the scores [41, 42]: food-based DHD15-index is conceptually related to food-based dietary guidelines and easily captures intakes of nutrient-dense plant-based foods versus animal-based foods; while the nutrient-based NRD9.3 evaluates dietary quality based on nutrient intake relative to nutritional requirements irrespective of the food sources. A sole focus on food-based approaches to a healthy and environmentally-friendly diet may therefore not capture the full spectrum of nutritional risks and may incorrectly lump all sustainability indicators together. Research is still needed to identify appropriate diet scores, differentially weighing various aspects of healthy and environmentally-friendly diets [43].

## Conclusion

In conclusion, estimations of the environmental impact of the diet are dependent of the method of dietary assessment; the FFQ slightly underestimated environmental impact when compared with the 24hR. Using energy-adjusted values resulted in a higher group mean bias and a lower correlation between FFQ and 24hR, but there was less attenuation. Correlation coefficients and attenuation coefficients for environmental impact measures behaved in a similar way as for protein intake, this suggests that our findings and conclusions related to covariate- and energy-adjustment can be extended to other dietary factors. Moreover, de-attenuation of the 24hR and calibration of the FFQ to 24hR increases the strength of the associations between dietary quality and diet-related environmental impact. Higher dietary quality was associated with improved environmental impact for food-based scores, but no clear associations for nutrient-based scores. It is therefore important to include nutrient-based approaches, next to food-based approaches, to prevent that the transition to environmentally-friendly diets negatively affects nutritional status of the population.

## Additional files

Additional file 1: **Table S1.** Diet-related environmental impact according to the food frequency questionnaire (FFQ) and two replicates of the 24-h recall (24hR, with intra-class correlation coefficient) and group level bias, with correlation between the methods (crude, adjusted, de-attenuated) and attenuation coefficient (crude, adjusted) for observed and energy-adjusted values standardised to a 2000 kcal diet in men^a^. (DOCX 34 kb)

Additional file 2: **Table S2.** Diet-related environmental impact according to the food frequency questionnaire (FFQ) and two replicates of the 24-h recall (24hR, with intra-class correlation coefficient) and group level bias, with correlation between the methods (crude, adjusted, de-attenuated) and attenuation coefficient (crude, adjusted) for observed and energy-adjusted values standardised to a 2000 kcal diet in women^a^. (DOCX 34 kb)
