# Supplementary material for: FFQ versus repeated 24-h recalls for estimating diet-related environmental impact
Source: Nutr J. 2019 Jan 8;18:2. doi: 10.1186/s12937-018-0425-z (PMC6323679; doi:10.1186/s12937-018-0425-z)
Supplement: Supplementary file 1 — Table S1. Diet-related environmental impact according to the food frequency questionnaire (FFQ) and two replicates of the 24-h recall (24hR, with intra-class correlation coefficient) and group level bias, with correlation between the methods (crude, adjusted, de-attenuated) and attenuation coefficient (crude, adjusted) for observed and energy-adjusted values standardised to a 2000 kcal diet in mena. (DOCX 34 kb) [file 12937_2018_425_MOESM1_ESM.docx]

**Table S1** Diet-related environmental impact according to the food frequency questionnaire (FFQ) and two replicates of the 24-hour recall (24hR, with intra-class correlation coefficient) and group level bias, with correlation between the methods (crude, adjusted, de-attenuated) and attenuation coefficient (crude, adjusted) for observed and energy-adjusted values standardised to a 2,000 kcal diet in men ^a^.

|  | FFQ | | 2 replicates of 24hR | | | | Correlation coefficient (24hR with FFQ) | | | | | | Attenuation coefficient λ_1_ | | | |
| --- | --- | --- | --- | --- | --- | --- | --- | --- | --- | --- | --- | --- | --- | --- | --- | --- |
| Dietary variables | Mean | (SD) | Mean | (SD) | ICC | %bias | Crude (95%CI) | | Adjusted ^b^(95%CI) | | De-attenuated (95%CI) | | Crude (SE) | | Adjusted ^b^ (SE) | |
| *Observed values* | | | | | | | | | | | | | | | | |
| Energy, kcal/d | 2348 | (534) | 2200 | (617) | 0.31 | 6.7 | 0.42 | (0.35; 0.48) | 0.39 | (0.32; 0.45) | 0.69 | (0.57; 0.81) | 0.49 | (0.04) | 0.45 | (0.04) |
| Protein, g/d | 83.8 | (18.8) | 84.9 | (24.8) | 0.27 | -1.3 | 0.41 | (0.34; 0.47) | 0.39 | (0.32; 0.45) | 0.74 | (0.61; 0.87) | 0.54 | (0.05) | 0.51 | (0.05) |
| GHGE, kgCO_2_e/d | 3.77 | (0.88) | 3.94 | (1.60) | 0.18 | -4.3 | 0.28 | (0.21; 0.35) | 0.27 | (0.20; 0.34) | 0.64 | (0.46; 0.81) | 0.51 | (0.07) | 0.50 | (0.07) |
| FE, MJ/d | 32.40 | (6.89) | 33.36 | (9.83) | 0.28 | -2.9 | 0.40 | (0.33; 0.47) | 0.40 | (0.33; 0.46) | 0.75 | (0.61; 0.87) | 0.57 | (0.05) | 0.57 | (0.05) |
| LU, m^2^*year/d | 4.38 | (1.06) | 4.57 | (1.99) | 0.18 | -4.2 | 0.29 | (0.21; 0.36) | 0.28 | (0.20; 0.35) | 0.67 | (0.49; 0.84) | 0.54 | (0.07) | 0.52 | (0.07) |
| pReCiPe | 0.45 | (0.10) | 0.46 | (0.18) | 0.19 | -2.2 | 0.31 | (0.23; 0.38) | 0.30 | (0.22; 0.37) | 0.68 | (0.51; 0.84) | 0.54 | (0.07) | 0.53 | (0.07) |
| *Energy-adjusted values by regression residuals of observed values on energy(observed residuals)* | | | | | | | | | | | | | | | | |
| Protein, g | 73.8 | (11.0) | 78.6 | (15.1) | 0.19 | -6.1 | 0.41 | (0.34; 0.47) | 0.31 | (0.23; 0.38) | 0.70 | (0.53; 0.86) | 0.44 | (0.05) | 0.42 | (0.05) |
| GHGE, kgCO_2_e | 3.37 | (0.64) | 3.69 | (1.40) | 0.12 | -8.7 | 0.28 | (0.21; 0.35) | 0.21 | (0.13; 0.29) | 0.61 | (0.39; 0.82) | 0.51 | (0.09) | 0.48 | (0.09) |
| FE, MJ | 28.93 | (4.35) | 31.23 | (7.19) | 0.20 | -7.4 | 0.38 | (0.31; 0.44) | 0.36 | (0.28; 0.42) | 0.80 | (0.64; 0.95) | 0.62 | (0.06) | 0.61 | (0.07) |
| LU, m^2^*year | 3.88 | (0.72) | 4.26 | (1.74) | 0.12 | -8.9 | 0.26 | (0.19; 0.33) | 0.24 | (0.16; 0.32) | 0.69 | (0.47; 0.90) | 0.63 | (0.09) | 0.60 | (0.10) |
| pReCiPe, | 0.40 | (0.07) | 0.43 | (0.15) | 0.13 | -7.0 | 0.27 | (0.19; 0.34) | 0.25 | (0.17; 0.32) | 0.68 | (0.47; 0.88) | 0.56 | (0.09) | 0.57 | (0.09) |
| *Energy-adjusted values by regression residuals of densities on energy (density residuals)* | | | | | | | | | | | | | | | | |
| Protein density, % | 14.8 | (1.9) | 16.0 | (2.85) | 0.18 | -7.5 | 0.32 | (0.25; 0.39) | 0.31 | (0.23; 0.38) | 0.73 | (0.55; 0.89) | 0.48 | (0.06) | 0.46 | (0.06) |
| GHGEdensity,kgCO_2_e | 3.39 | (0.55) | 3.75 | (1.28) | 0.16 | -9.6 | 0.25 | (0.17; 0.32) | 0.22 | (0.15; 0.30) | 0.57 | (0.37; 0.75) | 0.57 | (0.09) | 0.53 | (0.09) |
| FE density, MJ | 29.10 | (3.77) | 32.07 | (6.63) | 0.21 | -9.3 | 0.39 | (0.32; 0.46) | 0.37 | (0.30; 0.44) | 0.80 | (0.65; 0.94) | 0.69 | (0.07) | 0.66 | (0.07) |
| LU density, m^2^*year | 3.90 | (0.65) | 4.33 | (1.62) | 0.13 | -9.9 | 0.27 | (0.19; 0.34) | 0.24 | (0.17; 0.32) | 0.67 | (0.46; 0.88) | 0.66 | (0.10) | 0.62 | (0.10) |
| pReCiPe density | 0.40 | (0.06) | 0.44 | (0.14) | 0.17 | -9.1 | 0.28 | (0.20; 0.35) | 0.26 | (0.18; 0.33) | 0.63 | (0.44; 0.81) | 0.65 | (0.09) | 0.61 | (0.09) |

ICC, intra class correlation coefficient; GHGE, greenhouse gas emissions; FE, fossil energy use; LU, land use; pReCiPe, a weighted summary score for GHGE, FE, and LU; % bias, group-level bias calculated as (mean intake FFQ /mean intake 24hR)x100; 100; correlation coefficient (95%CI) estimated as the Pearson correlation coefficient; de-attenuated correlation coefficient (95%CI) estimated as the Pearson correlation coefficient/√ICC_24hR_; Attenuation coefficient λ_1_ (SE) estimated as the slope in the linear regression of the 24hR on the FFQ using linear mixed models to account for within-person day-to-day variability.

^a^ Mean values with their standard deviations, correlation coefficient with its 95% confidence intervals, attenuation coefficient with its standard error.

^b^ Adjusted for age and BMI.
