# Supplementary material for: FFQ versus repeated 24-h recalls for estimating diet-related environmental impact
Source: Nutr J. 2019 Jan 8;18:2. doi: 10.1186/s12937-018-0425-z (PMC6323679; doi:10.1186/s12937-018-0425-z)
Supplement: Supplementary file 2 — Table S2. Diet-related environmental impact according to the food frequency questionnaire (FFQ) and two replicates of the 24-h recall (24hR, with intra-class correlation coefficient) and group level bias, with correlation between the methods (crude, adjusted, de-attenuated) and attenuation coefficient (crude, adjusted) for observed and energy-adjusted values standardised to a 2000 kcal diet in womena. (DOCX 34 kb) [file 12937_2018_425_MOESM2_ESM.docx]

**Table S2** Diet-related environmental impact according to the food frequency questionnaire (FFQ) and two replicates of the 24-hour recall (24hR, with intra-class correlation coefficient) and group level bias, with correlation between the methods (crude, adjusted, de-attenuated) and attenuation coefficient (crude, adjusted) for observed and energy-adjusted values standardised to a 2,000 kcal diet in women^a^ .

|  | FFQ | | 2 replicates of 24hR | | | | | Correlation coefficient (24hR with FFQ) | | | | | | | Attenuation coefficient λ_1_ | | | | |
| --- | --- | --- | --- | --- | --- | --- | --- | --- | --- | --- | --- | --- | --- | --- | --- | --- | --- | --- | --- |
| Dietary variables | Mean | (SD) | Mean | (SD) | ICC | %bias | | Crude (95%CI) | | Adjusted ^b^(95%CI) | | | De-attenuated (95%CI) | | | Crude (SE) | | Adjusted ^b^ (SE) | |
| *Observed values* | | | | | | | | | | | | | | | | | | | |
| Energy, kcal/d | 1915 | (431) | 1808 | (466) | 0.29 | | 5.9 | 0.34 | (0.27; 0.41) | | 0.35 | (0.27; 0.42) | 0.64 | (0.50; 0.77) | | 0.37 | (0.04) | 0.37 | (0.04) |
| Protein, g/d | 70.9 | (15.6) | 71.0 | (19.7) | 0.27 | | -0.1 | 0.38 | (0.31; 0.45) | | 0.38 | (0.31; 0.45) | 0.74 | (0.60; 0.87) | | 0.48 | (0.05) | 0.49 | (0.05) |
| GHGE, kgCO_2_e/d | 3.20 | (0.76) | 3.32 | (1.20) | 0.14 | | -3.6 | 0.35 | (0.28; 0.42) | | 0.35 | (0.27; 0.42) | 0.92 | (0.72; 1.11) | | 0.55 | (0.06) | 0.56 | (0.07) |
| FE, MJ/d | 27.81 | (5.62) | 28.66 | (7.77) | 0.27 | | -3.0 | 0.39 | (0.32; 0.46) | | 0.39 | (0.32; 0.46) | 0.75 | (0.61; 0.0.88) | | 0.54 | (0.05) | 0.56 | (0.06) |
| LU, m^2^*year/d | 3.60 | (0.94) | 3.71 | (1.51) | 0.17 | | -3.0 | 0.37 | (0.30; 0.44) | | 0.36 | (0.29; 0.43) | 0.88 | (0.70; 1.05) | | 0.60 | (0.06) | 0.60 | (0.07) |
| pReCiPe | 0.37 | (0.09) | 0.39 | (0.14) | 0.18 | | -5.1 | 0.38 | (0.30; 0.44) | | 0.37 | (0.29; 0.44) | 0.88 | (0.70; 1.04) | | 0.58 | (0.06) | 0.59 | (0.06) |
| *Energy-adjusted values by regression residuals of observed values on energy(observed residuals)* | | | | | | | | | | | | | | | | | | | |
| Protein, g | 73.3 | (9.5) | 77.1 | (13.1) | 0.21 | | -4.9 | 0.38 | (0.31; 0.45) | | 0.37 | (0.30; 0.44) | 0.67 | (0.51; 0.82) | | 0.56 | (0.05) | 0.52 | (0.06) |
| GHGE, kgCO_2_e | 3.30 | (0.59) | 3.58 | (1.04) | 0.11 | | -7.8 | 0.35 | (0.28; 0.42) | | 0.26 | (0.18; 0.33) | 0.76 | (0.53; 0.99) | | 0.52 | (0.07) | 0.47 | (0.08) |
| FE, MJ | 28.58 | (4.03) | 30.57 | (6.27) | 0.24 | | -6.5 | 0.40 | (0.33; 0.46) | | 0.35 | (0.28; 0.42) | 0.71 | (0.56; 0.86) | | 0.62 | (0.06) | 0.57 | (0.06) |
| LU, m^2^*year | 3.73 | (0.69) | 4.01 | (1.34) | 0.15 | | -7.0 | 0.34 | (0.26; 0.41) | | 0.31 | (0.23; 0.38) | 0.80 | (0.60; 0.99) | | 0.66 | (0.08) | 0.61 | (0.08) |
| pReCiPe, | 0.39 | (0.06) | 0.42 | (0.12) | 0.15 | | -7.1 | 0.34 | (0.26; 0.41) | | 0.30 | (0.22; 0.37) | 0.77 | (0.57; 0.96) | | 0.61 | (0.07) | 0.56 | (0.08) |
| *Energy-adjusted values by regression residuals of densities on energy (density residuals)* | | | | | | | | | | | | | | | | | | | |
| Protein density, % | 14.8 | (2.0) | 15.7 | (3.3) | 0.21 | | -5.7 | 0.40 | (0.33; 0.47) | | 0.37 | (0.30; 0.44) | 0.82 | (0.66; 0.97) | | 0.66 | (0.06) | 0.62 | (0.07) |
| GHGEdensity,kgCO_2_e | 3.35 | (0.64) | 3.67 | (1.31) | 0.21 | | -8.7 | 0.31 | (0.24; 0.39) | | 0.28 | (0.20; 0.35) | 0.61 | (0.44; 0.78) | | 0.65 | (0.08) | 0.60 | (0.09) |
| FE density, MJ | 29.11 | (4.35) | 31.65 | (8.47) | 0.35 | | -8.0 | 0.39 | (0.32; 0.46) | | 0.35 | (0.27; 0.42) | 0.59 | (0.46; 0.70) | | 0.76 | (0.08) | 0.71 | (0.08) |
| LU density, m^2^*year | 3.77 | (0.75) | 4.10 | (1.81) | 0.27 | | -8.0 | 0.32 | (0.24; 0.39) | | 0.29 | (0.21; 0.36) | 0.55 | (0.40; 0.69) | | 0.77 | (0.10) | 0.71 | (0.10) |
| pReCiPe density | 0.39 | (0.07) | 0.43 | (0.15) | 0.29 | | -9.3 | 0.34 | (0.26; 0.41) | | 0.30 | (0.22; 0.37) | 0.56 | (0.41; 0.69) | | 0.75 | (0.09) | 0.69 | (0.09) |

ICC, intra class correlation coefficient; GHGE, greenhouse gas emissions; FE, fossil energy use; LU, land use; pReCiPe, a weighted summary score for GHGE, FE, and LU; % bias, group-level bias calculated as (mean intake FFQ /mean intake 24hR)x100; 100; correlation coefficient (95%CI) estimated as the Pearson correlation coefficient; de-attenuated correlation coefficient (95%CI) estimated as the Pearson correlation coefficient/√ICC_24hR_; Attenuation coefficient λ_1_ (SE) estimated as the slope in the linear regression of the 24hR on the FFQ using linear mixed models to account for within-person day-to-day variability.

^a^ Mean values with their standard deviations, correlation coefficient with its 95% confidence intervals, attenuation coefficient with its standard error.

^b^ Adjusted for age and BMI.
